# Effects of increasing dietary inclusion of camelina cake on growth performance of growing-finishing pigs^1^

**DOI:** 10.1093/tas/txab140

**Published:** 2021-08-26

**Authors:** A M Hilbrands, L J Johnston, R B Cox, F Forcella, R Gesch, Y Z Li

**Affiliations:** 1 West Central Research and Outreach Center, University of Minnesota, Morris, MN, 56267, USA; 2 Department of Animal Science, University of Minnesota, St. Paul, MN 55108, USA; 3 USDA-ARS North Central Soil Conservation Research Lab, Morris, MN 56267, USA

**Keywords:** camelina, feed, growth performance, nutrition, pig, swine

## Abstract

The objective of this experiment was to determine the dietary inclusion rate of camelina cake (CC) that would support the growth performance of growing-finishing pigs similar to that of a corn-soybean meal-based diet. Pigs (*n* = 192; BW = 35.2 kg; Duroc x (Yorkshire x Landrace)), balanced for sex and initial weight, were assigned to pens (8 pigs/pen) and pens were assigned randomly to one of four dietary treatments (6 pens/treatment). Treatments consisted of a non GMO corn-soybean meal control diet (**CON**), or CON containing 5% (**5CC**), 10% (**10CC**), or 15% (**15CC**) camelina cake. Feed disappearance on a pen basis and individual body weights of pigs were recorded every other week to calculate average daily gain (ADG), average daily feed intake (ADFI), and gain to feed ratio (G:F) on a pen basis. Prior to harvest, real-time ultrasonic measurements of back fat depth and loin eye area were collected on all live pigs. Pigs were harvested as a single group at about 23 weeks of age at a commercial abattoir. Data were analyzed using Proc Glimmix with dietary treatment as a fixed effect and pen serving as the experimental unit. Growth performance data collected over time were analyzed using repeated measures within the Proc Glimmix procedure. Overall, pigs fed CON exhibited similar ADG to those consuming 5CC and higher ADG than pigs consuming 10CC and 15CC diets (1.10 kg vs. 1.05 kg for 10CC and 1.02 kg for 15CC; *P* < 0.05 for both mean comparisons). Pigs fed CON consumed more feed than pigs fed any of the CC diets (ADFI = 2.66 kg for CON vs. 2.46 kg for 5CC, 2.46 kg for 10CC and 2.47 kg for 15CC; *P* < 0.05 for all). These differences resulted in heavier (*P* < 0.05) CON-fed pigs at marketing than 10CC or 15CC-fed pigs. There were no differences in any carcass traits analyzed. From these data, we conclude that feeding up to 5% CC in corn-soybean meal-based diets did not negatively influence growth performance, or carcass traits of growing-finishing pigs.

## INTRODUCTION

Increased awareness of soil health and fertility coupled with the push to improve land productivity has intensified the adoption of cover crops in agricultural systems (Myers et al., 2019). Traditionally, cover crops were grown primarily for their benefit to the soil rather than their potential crop yield. With volatile commodity prices and unstable grain markets, producers have become interested in added-value cover crops that benefit soil health and fertility but can also be harvested for additional revenue (SARE, 2012; Imerman and Imerman, 2019). Camelina [*Camelina sativa* (L.) Crantz] is an oilseed crop belonging to the Brassicaceae family that adapts easily to diverse climatic conditions, has low nutrient requirements, and is resistant to many pests and diseases (Berti et al., 2016; Murphy, 2016). Gesch et al. (2014) successfully incorporated camelina in a relay cropping system with soybean [*Glycine max*. (L.) Merr.] as far north as Minnesota. Various cultivars of camelina exist with seeds typically containing between 35% and 45% oil (Przybylski, 2005; Graham et al., 2013; Berti et al., 2016). Camelina is processed by mechanical pressing, solvent extraction, or by a newer method which utilizes carbon dioxide under supercritical conditions (Matthäus, 2012)_._ Camelina oil can be used as an edible oil or in the biodiesel production. Material remaining after oil extraction is a residual cake with potential use as livestock feed. Camelina cake tends to have higher protein (Plessers et al., 1962) and amino acid concentrations (Zubr, 1997; Pekel et al., 2009) than meal resulting from either rape (*Brassica napus* L.) or flax (*Linum usitatissimum* L.) seed. Almeida et al. (2013) indicated digestibility of crude protein and amino acids in expeller-processed camelina cake when fed to pigs is similar to that of canola meal. Graham et al. (2013) reported decreased SID amino acid availability and lower nutrient concentration for camelina cake when compared with soybean meal. Antinutritional factors commonly found in seeds of plants belonging to the *Brassica* family can have negative impacts on digestibility and feed intake (Zubr, 1997; Schuster and Friedt, 1998; Matthäus and Angelini, 2005). Growing conditions, oil extraction methods, and variety can affect the level of antinutritional factors present in camelina cake. We suspected that increasing concentrations of camelina cake in pig diets would lead to decreased performance; therefore, the objective of this experiment was to determine what concentration of camelina cake could be fed in diets without depressing the growth performance of growing-finishing pigs. Our null hypothesis was that camelina cake would not affect pig performance.

## MATERIALS AND METHODS

The experimental protocol used in this study was approved by the University of Minnesota Institutional Animal Care and Use Committee.

### Animals and Housing

This experiment was conducted in the swine research unit at the University of Minnesota’s West Central Research and Outreach Center (Morris, MN). Pigs were housed in an environmentally controlled, growing-finishing barn with a target room temperature of 20˚C. Each pen (1.60 × 4.5 m) was equipped with 2 nipple waterers, one 4-space self-feeder, and totally slatted, concrete floors. All diets met or exceeded NRC (2012) nutrient requirements for growing-finishing pigs gaining 350 g lean/day. Pigs were allowed ad libitum access to feed and water throughout the experiment. Pigs were Duroc sired terminal offspring of Yorkshire x Landrace sows (Topigs Norsvin, Burnsville, MN).

Eleven week old pigs (*n* = 192; body weight (BW) = 35.3 kg) balanced for sex and weight were housed 8 pigs/pen and assigned randomly to 1 of 24 pens (6 pens/treatment). Sex ratio (5 barrows and 3 gilts) within pens was kept consistent among pens. Pens were assigned randomly to one of four dietary treatments fed in four phases.

### Dietary Treatments

Non genetically modified (GMO) corn (Midwest Protein, Grove City, MN), soybean meal (South Dakota Soybean Processors - Miller, St. Lawrence, SD), and camelina cake (Linnaeus Plant Sciences Inc., Surrey, BC. Canada V3Z 6R9) were sourced in single lots. Spring camelina (variety *Midas*) was used to produce camelina cake through hot-pressing for the study. Corn, soybean meal, and camelina cake were analyzed by Midwest Laboratories (Omaha, NE) to determine concentrations of crude protein (method 990.03), calcium and phosphorus (method 985.01 (mod)), crude fat (method 954.02 (mod)), crude fiber (method Ba 6a 05) according to methods described by AOAC (2006; Table 1). Amino acid profiles (method 982.30 E (a, b, c); AOAC, 2006) were analyzed at University of Missouri’s Agricultural Experiment Station Chemical Laboratory (Columbia, MO). Diets were formulated using these values along with amino acid digestibilities reported by NRC (2012) for corn and soybean meal. Amino acid digestibilities for camelina expeller 2 reported by Almeida et al. (2013) were used to formulate diets containing camelina cake. Experimental diets (Tables 2 and 3) consisted of a non GMO corn-soybean meal control diet (**CON**), or CON containing 5% (**5CC**), 10% (**10CC**), or 15% (**15CC**) camelina cake. Diets were fed in four phases based on BW (Phase 1: 23–50 kg, Phase 2: 50–75 kg, Phase 3: 75–100 kg and Phase 4: 100 kg to market). Phase changes were made when average BW of pigs within a pen was within 2.3 kg of the targeted beginning weight of the next phase. All pigs consumed a standard corn-soybean meal diet prior to the beginning of the experiment. No feed or water medication or growth-promoting additives were used throughout the study. Subsamples of a phase one diet from each treatment were obtained by the quartering method and analyzed (BioProfile Testing Labs, LLC., St. Paul, MN) for glucosinolate (method AK-92; AOAC, 2006) and trypsin inhibitor (method BA 12-75; AOAC, 2006) concentrations.

**Table 1.** Analyzed nutrient and amino acid content of dietary ingredients^1^,^2^

	Camelina cake	Soybean meal	Corn
Crude protein^3^, %	33.1	47.2	7.18
Crude Fat, %	13.9	5.38	3.18
Crude Fiber, %	9.98	2.02	1.5
Lysine	1.25	2.95	0.26
Threonine	1.24	1.80	0.24
Methionine	0.56	0.65	0.12
Cysteine	0.65	0.67	0.14
Tryptophan	0.41	0.75	0.06
Isoleucine	1.33	2.28	0.26
Valine	1.68	2.34	0.34
Arginine	2.39	3.36	0.32
Histidine	0.67	1.23	0.20
Leucine	2.04	3.61	0.82
Phenylalanine	1.44	2.44	0.35
Tyrosine	0.87	1.73	0.20
Taurine	0.06	0.06	0.09
Hydroxyproline	0.30	0.06	0.02
Aspartic Acid	2.62	5.26	0.47
Serine	1.21	1.93	0.31
Glutamic Acid	5.20	8.31	1.23
Proline	1.53	2.45	0.58
Lanthionine	0.08	0.06	0.00
Glycine	1.66	1.97	0.26
Alanine	1.44	2.00	0.50
Hydroxylysine	0.05	0.07	0.02
Ornithine	0.05	0.05	0.00

^1^W/W% = grams per 100 grams of sample.

^2^Corn, soybean meal, and camelina cake were analyzed by Midwest Laboratories (Omaha, NE) to determine concentrations of crude protein (method 990.03), calcium and phosphorus (method 985.01 (mod)), crude fat (method 954.02 (mod)), crude fiber (method Ba 6a 05) according to methods described by AOAC (2006). Amino acid profiles (method 982.30 E (a, b, c); AOAC, 2006) were analyzed at University of Missouri’s Agricultural Experiment Station Chemical Laboratory (Columbia, MO).

^3^Crude protein = %*N* × 6.25.

**Table 2.** Ingredient composition and calculated nutrient contentof experimental diets (as-fed basis), phases 1 and 2.

	Phase 1 (23–50 kg BW)				Phase 2 (50–75 kg BW)			
Ingredient, %	CON^1^	5CC^1^	10CC^1^	15CC^1^	CON	5CC	10CC	15CC
Corn	63.10	59.96	56.78	53.57	69.35	66.20	63.11	59.90
Soybean meal	34.32	32.52	30.72	28.93	28.25	26.45	24.64	22.85
Camelina meal	0.0	5.00	10.00	15.00	0.0	5.00	10.00	15.00
Monocalcium phosphate	1.07	1.04	1.03	1.03	0.93	0.97	0.93	0.93
Limestone	1.00	0.98	0.97	0.97	0.87	0.91	0.87	0.87
Salt	0.33	0.32	0.32	0.32	0.28	0.30	0.29	0.29
Vitamin-TM premix^2^	0.18	0.18	0.18	0.18	0.16	0.17	0.16	0.16
Total	100.00	100.00	100.00	100.00	100.00	100.00	100.00	100.00
Calculated composition:								
DM, %	90.1	90.1	90.1	90.1	89.8	89.8	89.8	89.8
CP, %	21.7	22.2	22.8	23.3	19.3	19.8	20.4	20.9
Ca, %	0.68	0.68	0.69	0.70	0.62	0.62	0.62	0.63
P, %	0.63	0.65	0.68	0.71	0.59	0.61	0.63	0.66
Available P, %	0.28	0.28	0.29	0.31	0.27	0.28	0.28	0.29
ME, kcal/kg	3,274	3,256	3,234	3,214	3,287	3,267	3,250	3,228
ADF, %	3.6	4.1	4.6	5.1	3.5	4.0	4.4	4.9
NDF, %	8.6	9.2	9.9	10.6	8.6	9.3	10.0	10.6
Crude fat, %	4.0	4.4	4.7	5.0	3.9	4.3	4.6	4.9
SID Lys, %	1.03	1.03	1.03	1.03	0.88	0.88	0.88	0.88
SID Thr, %	0.68	0.69	0.69	0.69	0.60	0.60	0.61	0.61
SID Met + Cys, %	0.60	0.60	0.61	0.62	0.55	0.55	0.56	0.56
SID Trp, %	0.24	0.24	0.24	0.24	0.20	0.20	0.20	0.20

^1^Diets: CON = corn-soybean meal control; 5CC = diet containing 5% camelina cake (CC); 10CC = diet containing 10% CC; 15CC = diet containing 15% CC.

^2^Vitamin and trace mineral premix supplied the following per kilogram of premix: vitamin A, 3,527,360 IU; vitamin D_3_, 661,380 IU; vitamin E, 13,228 IU; vitamin K, 1,323 mg; riboflavin, 2,205 mg; niacin, 13,228 mg; pantothenic acid, 8,818 mg; vitamin B_12_, 13.3 mg; iodine as ethylenediamine dihydroiodide, 119.0 mg; selenium as sodium selenite, 119.0 mg; zinc as polysaccharide complex of zinc, 22,046 mg; iron as polysaccharide complex of iron, 13,228 mg; manganese as polysaccharide complex of manganese, 2,205 mg; and copper as polysaccharide complex of copper, 1,543 mg.

**Table 3.** Ingredient composition and calculated nutrient content of experimental diets (as-fed basis), phases 3 and 4.

Ingredient, %	Phase 3 (75 to 100 kg BW)				Phase 4 (100 kg BW to market)			
	CON^1^	5CC^1^	10CC^1^	15CC^1^	CON	5CC	10CC	15CC
Corn	74.28	70.72	67.15	63.57	78.59	75.03	71.43	67.89
Soybean meal	23.50	22.15	20.79	19.43	19.38	18.02	16.67	15.31
Camelina meal	0.0	5.00	10.00	15.00	0.0	5.00	10.00	15.00
Monocalcium phosphate	0.92	0.89	0.85	0.83	0.84	0.80	0.79	0.74
Limestone	0.86	0.83	0.80	0.78	0.79	0.76	0.74	0.70
Salt	0.28	0.27	0.26	0.25	0.26	0.25	0.24	0.23
Vitamin-TM premix^2^	0.16	0.15	0.15	0.14	0.14	0.14	0.13	0.13
Total	100.00	100.00	100.00	100.00	100.00	100.00	100.00	100.00
Calculated composition:								
DM, %	86.8	87.1	87.4	87.7	86.5	86.8	87.1	87.4
CP, %	16.2	17.0	17.7	18.5	14.6	15.3	16.1	16.8
Ca, %	0.55	0.55	0.55	0.55	0.50	0.50	0.51	0.50
P, %	0.54	0.57	0.59	0.61	0.51	0.53	0.56	0.58
Available P, %	0.29	0.30	0.30	0.30	0.27	0.27	0.28	0.28
ME, kcal/kg	3,084	3,075	3,069	3,060	3,109	3,102	3,093	3,086
ADF, %	3.4	3.9	4.4	4.9	3.3	3.8	4.3	4.8
NDF, %	8.7	9.4	10.0	10.7	8.8	9.4	10.1	10.7
Crude fat, %	3.7	4.2	4.7	5.2	3.6	4.1	4.6	5.1
SID Lys, %	0.76	0.76	0.76	0.76	0.66	0.66	0.66	0.66
SID Thr, %	0.50	0.51	0.52	0.53	0.45	0.45	0.46	0.47
SID Met + Cys, %	0.43	0.44	0.46	0.47	0.39	0.41	0.42	0.44
SID Trp, %	0.19	0.19	0.19	0.20	0.17	0.17	0.17	0.17

^1^Diets: CON = corn-soybean meal control; 5CC = diet containing 5% camelina cake (CC); 10CC = diet containing 10% CC; 15CC = 15% CC.

^2^Vitamin and trace mineral premix supplied the following per kilogram of premix: vitamin A, 3,527,360 IU; vitamin D_3_, 661,380 IU; vitamin E, 13,228 IU; vitamin K, 1,323 mg; riboflavin, 2,205 mg; niacin, 13,228 mg; pantothenic acid, 8,818 mg; vitamin B_12_, 13.3 mg; iodine as ethylenediamine dihydroiodide, 119.0 mg; selenium as sodium selenite, 119.0 mg; zinc as polysaccharide complex of zinc, 22,046 mg; iron as polysaccharide complex of iron, 13,228 mg; manganese as polysaccharide complex of manganese, 2,205 mg; and copper as polysaccharide complex of copper, 1,543 mg.

### Experimental Procedure

Body weights of individual pigs were measured every other week, averaged within pen and used to calculate average daily gain (ADG) on a pen basis. Feed deliveries were also recorded by pen with feed disappearances measured on weigh days and used to calculate average daily feed intake (ADFI) on a pen basis. Gain efficiency (G:F) was calculated as ADG/ADFI on a pen basis.

At about 22 weeks of age, real-time ultrasound imaging (Exago model, Echo Control Medical, Angouleme, France) by a trained and certified technician was used to collect cross-sectional images at the 10th/11th rib interface on all live pigs. Back fat depth and loin muscle area at the 10th rib were computed from captured images using Biosoft Toolbox II for swine Software (Version 2.5.0.6; Biotronics Inc., Ames, IA).

At about 23 weeks of age, 22 gilts (5 from CON, 6 from 5CC, 6 from 10CC, and 5 from 15CC), each from a different pen and closest to the pen average body weight, were harvested at the University of Minnesota abattoir and analyzed for meat quality. Meat quality analysis are reported in a companion paper (Zhu et al., 2021). Remaining pigs were harvested five days later at a commercial abattoir (Hormel Foods, Austin, MN). Hot carcass weights (HCW) were measured on all pigs directly after slaughter. Carcass dressing percentage was calculated using HCW and final BW at the research farm via the following formula: dressing percentage = (HCW (kg)/final BW (kg)) × 100. We used the NPPC equation to calculate lean gain per day for live pigs using data collected from real-time ultrasound (NPPC, 2000). Fat-free lean percentage of carcasses was calculated using the NPPC equation for unribbed carcasses that included 10th rib loin muscle area and back fat depth derived from real-time ultrasound and HCW (NPPC, 2000).

### Statistical Analyses

Data were analyzed in a randomized complete block design using the Glimmix procedure of SAS (SAS Inst. Inc., Cary, NC). The statistical model for initial and final body weight, overall growth performance and carcass traits included dietary treatment as a fixed effect and pen as the experimental unit. Repeated measures analysis was used to determine effects of dietary treatments on growth performance collected across time with dietary treatment, time and their interaction as fixed effects and pen serving as the experimental unit in the model. Unstructured covariance structures were used to model errors within experimental units across time. Akaike’s Information Criterion (**AIC**) was used to determine the most appropriate covariance structure for each variable. The model with the smallest AIC value was considered the best fit for the data and is reported here.

All means are reported as least square means. We performed linear contrasts for overall performance data within Proc Glimmix. Individual mean separations were accomplished using the PDIFF option of SAS with the Tukey–Kramer adjustment for multiple comparisons. Satterthwaite’s procedure was used to approximate denominator degrees of freedom. The significance level was set at *P <* 0.05, with 0.05 < *P <* 0.10 indicating a trend.

## RESULTS

During the experiment, one pig was removed from 5CC due to illness, one from 10CC due to death and one from 15CC due to chronic lameness. Five pigs were not marketed at the end of the experiment due to either being too light (<100 kg) for harvest at the commercial abattoir (1 pig from 5CC and 2 pigs from 10CC), lameness (1 pig from 15CC) or having an abdominal rupture (1 pig from 15CC).

Control pigs gained more (*P* < 0.002) weight per day than pigs consuming either the 10% or 15% camelina diets (Table 4). Pigs assigned to the control diet consumed more (*P* < 0.002) feed daily than those assigned to any of the camelina-containing diets. Higher feed intakes and body weight gains in the control pigs resulted in heavier (*P* = 0.003) final body weights than in pigs consuming the 10CC or 15CC diets. There were no differences in gain efficiency among CON pigs and pigs fed camelina diets. Inclusion of camelina cake had no effect on ultrasonic measurements of loin eye area or backfat depth. Pigs consuming the CON diet had heavier (*P* = 0.01) HCWs than pigs consuming 15CC. Dressing percent, fat-free lean in carcass, and fat-free lean gain per day were not affected by diet.

**Table 4.** Overall growth performance and carcass traits

Item	Dietary treatments^1^					
	CON	5CC	10CC	15CC	SE	P <^2^
Growth performance						
No. of pens	6	6	6	6		
No. of pigs	48	48	48	48		
Initial body weight, kg	35.2	35.4	35.3	35.1	0.32	0.91
ADG, kg	1.10^a^	1.06^ab^	1.05^b^	1.02^b^	0.012	0.001
ADFI, kg	2.66^a^	2.46^b^	2.46^b^	2.47^b^	0.037	0.002
Final body weight, kg	126.4^a^	123.4^ab^	122.2^b^	120.0^b^	1.06	0.002
Gain efficiency (G:F)	0.41	0.43	0.43	0.42	0.006	0.782
Ultrasound measurements^3^						
No. of pigs^4^	48	47	47	47		
Loin muscle area, cm^2^	41.0	40.4	40.1	39.8	0.58	0.21
10th rib backfat depth, mm	24.2	25.3	22.1	22.6	1.90	0.27
Carcass traits						
No. of pigs^5^	43	40	39	40		
Hot carcass weight, kg	97.1^a^	94.8^ab^	93.9^ab^	91.1^b^	1.32	0.01
Dressing percent	74.4	74.0	74.2	73.6	0.25	0.54
Fat-free lean, %	48.06	47.73	48.91	48.83	0.683	0.209
Fat-free lean gain per day, kg	0.40	0.38	0.39	0.38	0.007	0.307

^ab^Within a row, means without a common superscript differ (*P* < 0.05).

^1^CON = corn-soybean meal diet; 5CC = CON + 5% camelina cake (CC); 10CC = CON + 10% CC; 15CC = CON + 15% CC.

^2^Linear effect.

^3^Real-time ultrasound measurements recorded on live pigs at about 22 weeks of age.

^4^Pigs available for real-time ultrasonic imaging.

^5^Pigs harvested at commercial abattoir.

**Figure 1.**
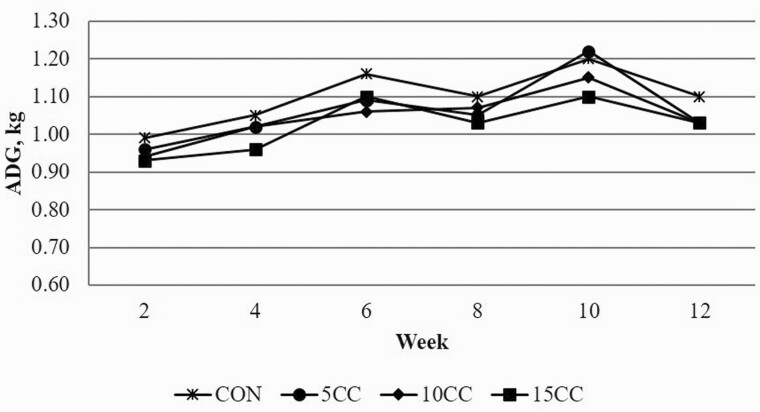
Effect of inclusion rate of camelina cake on ADG over time. CON = corn-soybean meal diet; 5CC = CON + 5% camelina cake (CC); 10CC = CON + 10% CC; 15CC = CON + 15% CC.

**Figure 2.**
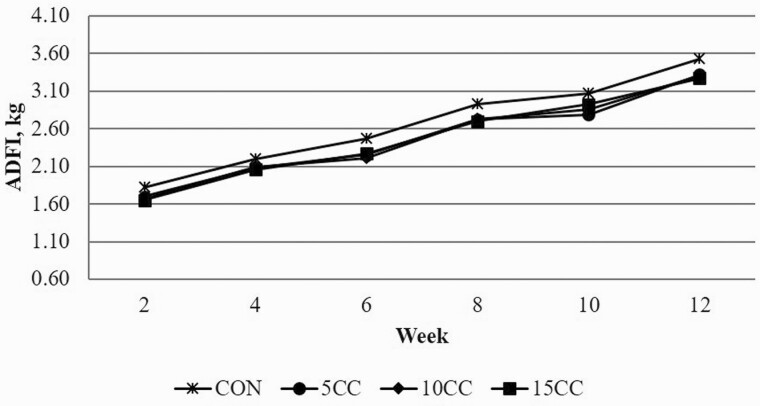
Effect of inclusion rate of camelina cake on ADFI over time. CON = corn-soybean meal diet; 5CC = CON + 5% camelina cake (CC); 10CC = CON + 10% CC; 15CC = CON + 15% CC.

**Figure 3.**
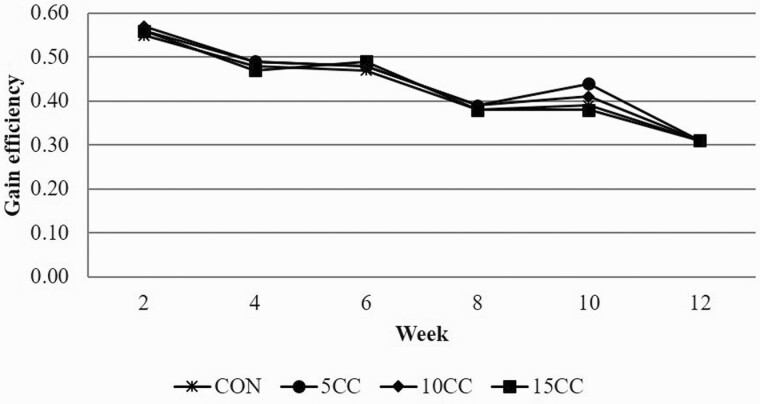
Effect of inclusion rate of camelina cake on gain efficiency over time. CON = corn-soybean meal diet; 5CC = CON + 5% camelina cake (CC); 10CC = CON + 10% CC; 15CC = CON + 15% CC.

Time trend analysis of ADG (Figure 1), ADFI (Figure 2), and G:F (Figure 3) support overall growth performance data reported in Table 4. No dietary treatment by time interactions were observed suggesting that pigs’ response to dietary camelina cake was consistent throughout the growing-finishing period.

## DISCUSSION

Nutrient concentrations of camelina cake can vary due to environmental factors and seed varieties. The crude protein concentration of the camelina cake used in our experiment was marginally lower than that reported by Nain et al. (2015) and Smit and Beltranena (2017a, 2017b), but similar with those reported by Almeida et al. (2013). Crude fiber level of our camelina cake was similar to that reported by Nain et al. (2015) and lower than those reported by Smit and Beltranena (2017a, 2017b). Crude fat levels in our camelina cake were similar to those reported by Smit and Beltranena (2017b) but higher than reported by Smit and Beltranena (2017a).

In our current study, CON pigs gained more weight per day than pigs consuming a diet containing 10% or 15% camelina cake. Low nutrient digestibility of camelina cake in experimental diets could lead to reductions in growth. Graham et al. (2013) reported decreased SID amino acid availability and lower nutrient concentration for camelina cake when compared with soybean meal. Kahindi et al. (2014) also reported low digestibility coefficients for screw-pressed camelina cake and noted differences in digestibility among camelina cake samples caused by variations in oil extraction methods. We observed no statistically significant differences in gain efficiency or daily fat-free lean gain between CON pigs and pigs fed camelina-containing diets leading us to believe that we properly accounted for the low nutrient digestibility of camelina cake in the formulation of experimental diets. The depressed growth rate and feed intake of pigs fed 10CC and 15CC diets was not likely caused by poor nutrient digestibility of camelina cake.

Smit and Beltranena (2017b) noted a linear decrease in final body weight, feed intake, and weight gain as dietary inclusion rate of camelina cake in pig diets increased. We also observed decreases in feed intake when pigs consumed camelina diets regardless of inclusion rate. Antinutritive compounds commonly found in most species of the brassicaceae family may negatively affect feed intake and consequently body weight gain. Trypsin inhibitors are one such antinutritive compound that occur naturally in plant seeds and bind to the pancreatic enzymes, trypsin and chymotrypsin, resulting in reduced digestion of amino acids (Jezierny et al., 2010). Additionally, trypsin inhibitors can lead to increased cholecystokinin production which inhibits feed intake (Ripken et al., 2015). Thus, trypsin inhibitors could lead to reductions in feed intake both through increased cholecystokinin production and reductions in amino acid digestibility (Woyengo et al., 2017). Trypsin inhibitors in our current diets ranged from 1.45 to 2.205 Trypsin Inhibitor Units (TIU) mg^−1^. Pigs can consume up to 3.0 TIU mg^−1^ with no detrimental effects on nutrient digestibility of diets or pig performance (Woyengo et al., 2017). Because the concentration of trypsin inhibitors present in our diets were lower than levels known to depress pig performance, it appears as though trypsin inhibitors did not play a meaningful role in reducing weight gain of camelina-fed pigs.

Glucosinolates are secondary plant metabolites and precursors to antinutritive compounds found in the brassicaceae family. They are not toxic themselves, but can be degraded to various toxic products (isothiocynate, nitrile, and thiocynate) through mastication, milling, or heating of seeds (Murphy, 2016; Woyengo et al., 2017). Smit and Beltranena (2017a) concluded that decreasing feed intake of pigs observed with increasing concentrations of dietary camelina cake may have resulted from high glucosinolate levels in their camelina source. Glucosinolate compounds, in high concentrations, can inhibit iodine uptake by the thyroid gland leading to reduced growth and possibly goiter. In research conducted by Schöne et al. (1996, 1997, 2001), increasing dietary concentrations of glucosinolates through the inclusion of rapeseed meal in growing pig diets led to heavier thyroid glands relative to body weight and reductions in serum thyroxine (T_4_) concentrations, indicating toxicity. Current recommendations are to maintain total glucosinolate levels below 2 µmol g^−1^ in swine diets to avoid negative effects (Schöne et al., 1997, Tripathi and Mishra, 2007, Almeida et al., 2013). Schöne et al. (1997, 2001) recommended iodine supplementation in the presence of glucosinolates to counteract their antagonistic effect on animals. Iodine was not supplemented in the current study. Glucosinolate concentrations in our experimental diets ranged from 0.05 to 2.08 µmol g^−1^. Most research studies reported to date that focused on effects of glucosinolates on pig performance have relied on feed ingredients other than camelina cake. Mathäus and Zubr (2000) suggested detrimental effects of glucosinolates from camelina oilseed cake are less than those from rapeseed products which agrees with research reported by Almeida et al. (2013).

Based on measured levels of trypsin inhibitors and glucosinolates present in our experimental diets and data reported in scientific literature, the camelina diets should not have negatively affected pig performance in our current study. However, a group of focal pigs from this study, selected for further meat quality analysis and reported in a companion paper (Zhu et al., 2021), showed a significant increase in liver weight as a percentage of body weight when pigs consumed 10CC or 15CC compared with CON-fed pigs. This is in agreement with research reported by Schöne et al. (2001). Livers of 15CC pigs were heavier (*P* < 0.10) than those of CON-fed pigs. This observation suggests mild toxicity caused by the inclusion of camelina cake in pig diets. Based on these findings, we do believe there was a slight toxic effect of camelina cake leading to increased liver size and depressed feed intakes in camelina-fed pigs.

From these data, we conclude that feeding up to 5% camelina cake in corn-soybean meal-based diets did not negatively influence growth performance or carcass traits of growing-finishing pigs when trypsin inhibitor and glucosinolate concentrations were maintained below recommended concentrations.
